# Di-μ-chlorido-bis­{[2-(benzyl­imino­meth­yl)pyridine-κ^2^ *N*,*N*′]chloridomercury(II)} dichloridomercury(II)

**DOI:** 10.1107/S1600536810050725

**Published:** 2010-12-08

**Authors:** Young-Inn Kim, Young-Kwang Song, Seong-Jae Yun, In-Chan Kim, Sung Kwon Kang

**Affiliations:** a Department of Chemistry Education and Interdisciplinary Program of Advanced Information and Display Materials, Pusan National University, Busan 609-735, Republic of Korea; b Department of Chemistry, Pusan National University, Busan 609-735, Republic of Korea; c Department of Chemistry, Chungnam National University, Daejeon 305-764, Republic of Korea

## Abstract

The Hg^II^ ion in the title centrosymmetric dinuclear complex, [Hg_2_Cl_4_(C_13_H_12_N_2_)_2_]·[HgCl_2_], adopts a distorted square-pyramidal geometry, being coordinated by the bis-chelating *N*-heterocyclic ligand, two bridging Cl atoms and one terminal Cl atom. One of the bridging Hg—Cl bonds [2.8428 (11) Å] is significantly longer than the other [2.5327 (10) Å]. In the crystal, there are weak π–π inter­actions [centroid–centroid distance = 3.630 (3) Å] between the aromatic rings of the discrete units. The HgCl_2_ adduct molecule is located on an inversion centre and has an Hg—Cl bond length of 2.2875 (11) Å.

## Related literature

For general background to luminescent mercury compounds, see: Elena *et al.* (2006 ▶); Durantaye *et al.* (2006 ▶); Fan *et al.* (2009 ▶); He *et al.* (2008 ▶). For syntheses and structures of Hg(II) complexes, see: Kim & Kang (2010 ▶); Kim *et al.* (2010 ▶).
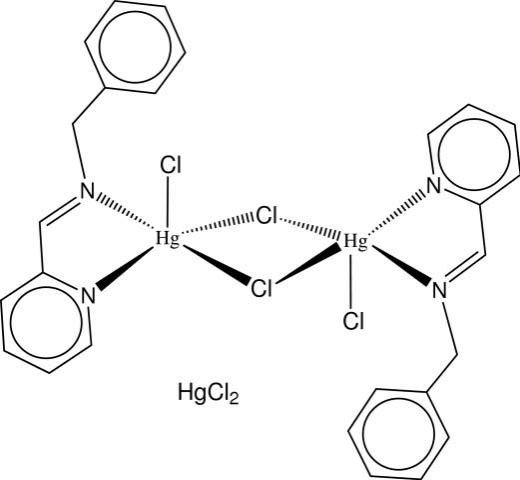


## Experimental

### 

#### Crystal data

[Hg_2_Cl_4_(C_13_H_12_N_2_)_2_]·[HgCl_2_]
*M* *_r_* = 1206.96
Monoclinic, 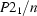

*a* = 10.1329 (2) Å
*b* = 8.1141 (1) Å
*c* = 19.0591 (2) Å
β = 92.939 (1)°
*V* = 1564.97 (4) Å^3^
*Z* = 2
Mo *K*α radiation
μ = 15.22 mm^−1^
*T* = 295 K
0.17 × 0.13 × 0.12 mm

#### Data collection

Bruker SMART CCD area-detector diffractometer
Absorption correction: multi-scan (*SADABS*; Bruker, 2002 ▶) *T*_min_ = 0.104, *T*_max_ = 0.158
16269 measured reflections
3892 independent reflections
3279 reflections with *I* > 2σ(*I*)
*R*_int_ = 0.027

#### Refinement

*R*[*F* ^2^ > 2σ(*F* ^2^)] = 0.025
*wR*(*F* ^2^) = 0.055
*S* = 1.04
3892 reflections
178 parameters
H-atom parameters constrained
Δρ_max_ = 1.21 e Å^−3^
Δρ_min_ = −1.18 e Å^−3^

### 

Data collection: *SMART* (Bruker, 2002 ▶); cell refinement: *SAINT* (Bruker, 2002 ▶); data reduction: *SAINT*; program(s) used to solve structure: *SHELXS97* (Sheldrick, 2008 ▶); program(s) used to refine structure: *SHELXL97* (Sheldrick, 2008 ▶); molecular graphics: *ORTEP-3 for Windows* (Farrugia, 1997 ▶); software used to prepare material for publication: *WinGX* (Farrugia, 1999 ▶).

## Supplementary Material

Crystal structure: contains datablocks global, I. DOI: 10.1107/S1600536810050725/jh2236sup1.cif

Structure factors: contains datablocks I. DOI: 10.1107/S1600536810050725/jh2236Isup2.hkl

Additional supplementary materials:  crystallographic information; 3D view; checkCIF report

## Figures and Tables

**Table:** 

Hg1—N8	2.347 (4)
Hg1—N1	2.373 (3)
Hg1—Cl1	2.4338 (12)

**Table:** 

N8—Hg1—N1	70.74 (12)
N8—Hg1—Cl1	113.97 (9)
N1—Hg1—Cl1	106.32 (9)
N8—Hg1—Cl2	95.92 (9)
N1—Hg1—Cl2	138.01 (8)
Cl1—Hg1—Cl2	115.34 (4)
N8—Hg1—Cl2^i^	142.19 (9)
N1—Hg1—Cl2^i^	83.87 (8)
Cl1—Hg1—Cl2^i^	99.58 (4)
Cl2—Hg1—Cl2^i^	84.37 (3)

Symmetry code: (i) 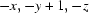.

**Fig. 1.**
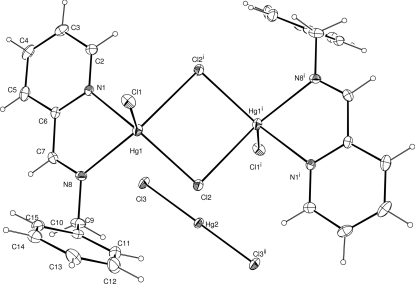
Molecular structure of (I), showing the atom-numbering scheme and 30% probability ellipsoids [symmetry code: (i) -x, -y + 1, -z; (ii) -x, -y + 2, -z].

## supplementary crystallographic information

### Comment

Much attention has been paid to the design and synthesis of luminescent mercury compounds for the detection and extraction of the mercury (Elena *et al.*, 2006; Durantaye *et al.*, 2006), among which, Hg(II) complexes with pyridine-containing ligands are of importance for their high luminescent efficiency (Fan *et al.*, 2009). In a previous report (Kim & Kang, 2010), we presented a structure of white Hg(II) complex with benzyl(2-pyridylmethylene)amine(bpma), (bpma)HgCl_2_, concerning its luminescence behavior (Kim *et al.*, 2010; He *et al.*, 2008). The reported white crystals were obtained after recrystallization from methanol solution in a day. However, we could find another yellow crystals in 3–4 days in the same solution. Herein, we report the structure of separated yellow crystals, [(bpma)HgCl_2_]_2_ HgCl_2_.

In (I), Fig. 1, the Hg1^II^ ion is coordinated by two N atoms of heterocyclic ligand, two bridging Cl atoms and one terminal Cl atom. The angles around Hg1 atoms are in the range of 70.74 (12) – 142.19 (9)°, suggesting the coordination geometry around the Hg1 atom is described as a distorted square pyrdmid with an apical position of Cl1 atom. One of the bridging Hg1—Cl bonds (2.843 (1) Å) is significantly longer than the other (2.533 (1) Å). The phenyl ring on the bpma ligand is twisted out of the pyridine plane, and form a dihedral angel of 81.21 (11)°. In the crystal structure, there are weak π-π interactions [centroid-centroid distance = 3.630 (3) Å] between the aromatic rings of the discrete units.

### Experimental

Benzyl(2-pyridylmethylene)amine (bpma) was synthesized from the reaction of 2-pyridinecarboxylaldehyde and benzylamine. And bpma reacted with mercury dichloride in methanol to yield the titled complex. The yellow crystals were separated from white crystals in 3–4 days from methanol solution. The detailed synthetic method was previously reported (Kim & Kang, 2010).

### Refinement

All H atoms were positioned geometrically and refined using a riding model, with C—H = 0.93 - 0.97 Å, and with *U*_iso_(H) = 1.2*U*_eq_(C). The maximum and minimum residual electron density peaks were located at 0.79 and 0.63 Å, respectively, from the Hg1 atom.

### Crystal data

**Table:**

[Hg_2_Cl_4_(C_13_H_12_N_2_)_2_]·[HgCl_2_]	*F*(000) = 1100
*M**_r_* = 1206.96	*D*_x_ = 2.561 Mg m^−^^3^
Monoclinic, *P*2_1_/*n*	Mo *K*α radiation, λ = 0.71073 Å
Hall symbol: -P 2yn	Cell parameters from 5840 reflections
*a* = 10.1329 (2) Å	θ = 2.2–28.0°
*b* = 8.1141 (1) Å	µ = 15.22 mm^−^^1^
*c* = 19.0591 (2) Å	*T* = 295 K
β = 92.939 (1)°	Block, yellow
*V* = 1564.97 (4) Å^3^	0.17 × 0.13 × 0.12 mm
*Z* = 2	

### Data collection

**Table:**

Bruker SMART CCD area-detector diffractometer	3279 reflections with *I* > 2σ(*I*)
φ and ω scans	*R*_int_ = 0.027
Absorption correction: multi-scan (*SADABS*; Bruker, 2002)	θ_max_ = 28.3°, θ_min_ = 2.1°
*T*_min_ = 0.104, *T*_max_ = 0.158	*h* = −13→10
16269 measured reflections	*k* = −10→10
3892 independent reflections	*l* = −25→25

### Refinement

**Table:**

Refinement on *F*^2^	0 restraints
Least-squares matrix: full	H-atom parameters constrained
*R*[*F*^2^ > 2σ(*F*^2^)] = 0.025	*w* = 1/[σ^2^(*F*_o_^2^) + (0.025*P*)^2^ + 1.1429*P*] where *P* = (*F*_o_^2^ + 2*F*_c_^2^)/3
*wR*(*F*^2^) = 0.055	(Δ/σ)_max_ < 0.001
*S* = 1.04	Δρ_max_ = 1.21 e Å^−^^3^
3892 reflections	Δρ_min_ = −1.18 e Å^−^^3^
178 parameters	

### Special details

**Table:**

Geometry. All e.s.d.'s (except the e.s.d. in the dihedral angle between two l.s. planes) are estimated using the full covariance matrix. The cell e.s.d.'s are taken into account individually in the estimation of e.s.d.'s in distances, angles and torsion angles; correlations between e.s.d.'s in cell parameters are only used when they are defined by crystal symmetry. An approximate (isotropic) treatment of cell e.s.d.'s is used for estimating e.s.d.'s involving l.s. planes.

### Fractional atomic coordinates and isotropic or equivalent isotropic displacement parameters (Å^2^)

**Table:**

	*x*	*y*	*z*	*U*_iso_*/*U*_eq_	
Hg1	0.092853 (16)	0.45122 (2)	0.092463 (9)	0.04770 (6)	
Cl1	0.02059 (13)	0.19456 (15)	0.14429 (6)	0.0625 (3)	
Cl2	−0.09059 (10)	0.64548 (14)	0.05096 (6)	0.0503 (3)	
N1	0.3257 (3)	0.4310 (4)	0.08642 (17)	0.0395 (7)	
C2	0.3903 (5)	0.3289 (6)	0.0459 (2)	0.0515 (11)	
H2	0.3421	0.2636	0.0137	0.062*	
C3	0.5252 (5)	0.3161 (7)	0.0498 (3)	0.0637 (14)	
H3	0.5671	0.2431	0.0206	0.076*	
C4	0.5977 (5)	0.4107 (8)	0.0965 (3)	0.0700 (17)	
H4	0.6895	0.4035	0.0997	0.084*	
C5	0.5319 (5)	0.5178 (7)	0.1392 (3)	0.0623 (14)	
H5	0.5789	0.5839	0.1716	0.075*	
C6	0.3952 (4)	0.5253 (5)	0.1331 (2)	0.0422 (9)	
C7	0.3196 (4)	0.6329 (5)	0.1779 (2)	0.0457 (10)	
H7	0.3644	0.7015	0.2101	0.055*	
N8	0.1950 (4)	0.6345 (4)	0.17363 (18)	0.0451 (8)	
C9	0.1223 (6)	0.7461 (6)	0.2188 (3)	0.0658 (14)	
H9A	0.0734	0.8259	0.1899	0.079*	
H9B	0.1844	0.8057	0.2498	0.079*	
C10	0.0277 (4)	0.6506 (6)	0.2621 (2)	0.0495 (10)	
C11	−0.1060 (5)	0.6560 (8)	0.2474 (3)	0.0740 (16)	
H11	−0.1404	0.7177	0.2096	0.089*	
C12	−0.1907 (5)	0.5681 (11)	0.2896 (3)	0.090 (2)	
H12	−0.2816	0.5723	0.28	0.108*	
C13	−0.1409 (6)	0.4767 (8)	0.3446 (3)	0.0747 (17)	
H13	−0.1975	0.4182	0.3724	0.09*	
C14	−0.0083 (6)	0.4711 (6)	0.3589 (3)	0.0625 (13)	
H14	0.0259	0.4081	0.3963	0.075*	
C15	0.0756 (5)	0.5576 (5)	0.3184 (3)	0.0531 (11)	
H15	0.1662	0.5537	0.329	0.064*	
Hg2	0	1	0	0.04776 (7)	
Cl3	0.21296 (11)	0.91503 (18)	0.02343 (7)	0.0664 (3)	

### Atomic displacement parameters (Å^2^)

**Table:**

	*U*^11^	*U*^22^	*U*^33^	*U*^12^	*U*^13^	*U*^23^
Hg1	0.03772 (9)	0.05296 (12)	0.05168 (11)	−0.00140 (7)	−0.00498 (7)	0.00188 (7)
Cl1	0.0824 (8)	0.0537 (7)	0.0518 (6)	−0.0145 (6)	0.0082 (6)	0.0031 (5)
Cl2	0.0455 (6)	0.0592 (7)	0.0456 (6)	0.0081 (5)	−0.0034 (4)	0.0008 (5)
N1	0.0362 (17)	0.0416 (19)	0.0408 (18)	−0.0014 (14)	0.0015 (14)	0.0033 (15)
C2	0.058 (3)	0.047 (3)	0.050 (3)	0.005 (2)	0.009 (2)	0.001 (2)
C3	0.063 (3)	0.062 (3)	0.068 (3)	0.018 (3)	0.024 (3)	0.020 (3)
C4	0.037 (2)	0.083 (4)	0.091 (4)	0.011 (3)	0.014 (3)	0.040 (3)
C5	0.046 (3)	0.069 (3)	0.071 (3)	−0.014 (2)	−0.012 (2)	0.021 (3)
C6	0.037 (2)	0.044 (2)	0.045 (2)	−0.0056 (16)	−0.0036 (17)	0.0101 (18)
C7	0.057 (3)	0.041 (2)	0.039 (2)	−0.0116 (19)	−0.0028 (18)	0.0030 (17)
N8	0.058 (2)	0.0355 (19)	0.0425 (19)	0.0052 (15)	0.0057 (16)	−0.0015 (14)
C9	0.094 (4)	0.043 (3)	0.063 (3)	0.011 (3)	0.020 (3)	−0.008 (2)
C10	0.056 (3)	0.045 (2)	0.048 (2)	0.010 (2)	0.007 (2)	−0.0124 (19)
C11	0.065 (3)	0.101 (4)	0.054 (3)	0.025 (3)	−0.008 (3)	−0.008 (3)
C12	0.043 (3)	0.154 (7)	0.071 (4)	−0.004 (3)	0.000 (3)	−0.034 (4)
C13	0.073 (4)	0.096 (5)	0.056 (3)	−0.025 (3)	0.013 (3)	−0.019 (3)
C14	0.073 (4)	0.055 (3)	0.060 (3)	−0.001 (2)	0.006 (3)	−0.006 (2)
C15	0.049 (3)	0.048 (3)	0.063 (3)	0.005 (2)	0.002 (2)	−0.009 (2)
Hg2	0.03219 (11)	0.05415 (15)	0.05669 (15)	0.00592 (9)	−0.00021 (10)	0.00053 (11)
Cl3	0.0374 (6)	0.0813 (9)	0.0799 (9)	0.0158 (6)	−0.0035 (5)	0.0004 (7)

### Geometric parameters (Å, °)

**Table:**

Hg1—N8	2.347 (4)	C7—H7	0.93
Hg1—N1	2.373 (3)	N8—C9	1.473 (5)
Hg1—Cl1	2.4338 (12)	C9—C10	1.510 (7)
Hg1—Cl2	2.5327 (10)	C9—H9A	0.97
Hg1—Cl2^i^	2.8428 (11)	C9—H9B	0.97
Cl2—Hg1^i^	2.8428 (11)	C10—C11	1.370 (7)
N1—C2	1.328 (5)	C10—C15	1.379 (6)
N1—C6	1.345 (5)	C11—C12	1.402 (9)
C2—C3	1.369 (6)	C11—H11	0.93
C2—H2	0.93	C12—C13	1.360 (10)
C3—C4	1.363 (8)	C12—H12	0.93
C3—H3	0.93	C13—C14	1.359 (8)
C4—C5	1.385 (8)	C13—H13	0.93
C4—H4	0.93	C14—C15	1.371 (7)
C5—C6	1.385 (6)	C14—H14	0.93
C5—H5	0.93	C15—H15	0.93
C6—C7	1.465 (6)	Hg2—Cl3^ii^	2.2875 (11)
C7—N8	1.262 (5)	Hg2—Cl3	2.2875 (11)
			
N8—Hg1—N1	70.74 (12)	N8—C7—H7	119.3
N8—Hg1—Cl1	113.97 (9)	C6—C7—H7	119.3
N1—Hg1—Cl1	106.32 (9)	C7—N8—C9	119.8 (4)
N8—Hg1—Cl2	95.92 (9)	C7—N8—Hg1	116.2 (3)
N1—Hg1—Cl2	138.01 (8)	C9—N8—Hg1	123.9 (3)
Cl1—Hg1—Cl2	115.34 (4)	N8—C9—C10	110.8 (4)
N8—Hg1—Cl2^i^	142.19 (9)	N8—C9—H9A	109.5
N1—Hg1—Cl2^i^	83.87 (8)	C10—C9—H9A	109.5
Cl1—Hg1—Cl2^i^	99.58 (4)	N8—C9—H9B	109.5
Cl2—Hg1—Cl2^i^	84.37 (3)	C10—C9—H9B	109.5
Hg1—Cl2—Hg1^i^	95.63 (3)	H9A—C9—H9B	108.1
C2—N1—C6	118.8 (4)	C11—C10—C15	118.8 (5)
C2—N1—Hg1	126.3 (3)	C11—C10—C9	121.4 (5)
C6—N1—Hg1	114.7 (3)	C15—C10—C9	119.8 (4)
N1—C2—C3	122.5 (5)	C10—C11—C12	119.6 (5)
N1—C2—H2	118.8	C10—C11—H11	120.2
C3—C2—H2	118.8	C12—C11—H11	120.2
C4—C3—C2	119.8 (5)	C13—C12—C11	120.4 (5)
C4—C3—H3	120.1	C13—C12—H12	119.8
C2—C3—H3	120.1	C11—C12—H12	119.8
C3—C4—C5	118.5 (5)	C14—C13—C12	119.8 (6)
C3—C4—H4	120.7	C14—C13—H13	120.1
C5—C4—H4	120.7	C12—C13—H13	120.1
C4—C5—C6	119.2 (5)	C13—C14—C15	120.4 (5)
C4—C5—H5	120.4	C13—C14—H14	119.8
C6—C5—H5	120.4	C15—C14—H14	119.8
N1—C6—C5	121.2 (4)	C14—C15—C10	120.9 (5)
N1—C6—C7	116.9 (4)	C14—C15—H15	119.5
C5—C6—C7	121.8 (4)	C10—C15—H15	119.5
N8—C7—C6	121.4 (4)	Cl3^ii^—Hg2—Cl3	180

Symmetry codes: (i) −*x*, −*y*+1, −*z*; (ii) −*x*, −*y*+2, −*z*.
